# Alignment of Midwifery Education in Nepal With Global Standards and Essential Competencies From the International Confederation of Midwives: A Mixed‐Methods Study

**DOI:** 10.1111/jmwh.70096

**Published:** 2026-04-06

**Authors:** Khim Bahadur Khadka, Deepak Paudel, Binod Bindu Sharma, Ramesh Prasad Adhikari, Anju Poudel, Urmila Baral, Kamala Rana Magar, Bidhya Banstola, Guenter Froeschl, Olena Ivanova

**Affiliations:** ^1^ Provincial Health Directorate Ministry of Health Pokhara Gandaki Nepal; ^2^ Center for International Health Ludwig‐Maximilians‐Universität Munich Germany; ^3^ Provincial Health Training Centre Ministry of Health Pokhara Gandaki Nepal; ^4^ Pokhara Academy of Health Sciences Western Regional Hospital Pokhara Nepal; ^5^ Institute of Infectious Diseases and Tropical Medicine University Hospital, Ludwig‐Maximilians‐Universität Munich Germany; ^6^ SPHERE National Health and Medical Research Council Centre of Research Excellence Melbourne Victoria Australia; ^7^ Department of General Practice, School of Public Health and Preventive Medicine Monash University Melbourne Victoria Australia

**Keywords:** curriculum, education, health care workforce, maternal and newborn health, midwifery, Nepal

## Abstract

**Introduction:**

Maternal and neonatal mortality rates in Nepal remain high, reflecting persistent gaps in the quality of maternal and newborn care. To address this issue, a national midwifery education program was initiated in 2016. This study assessed Nepal's preservice midwifery curricula against global standards and competencies defined by the International Confederation of Midwives (ICM).

**Methods:**

A mixed‐methods study was conducted, comprising a desk review of national standards and curricula against ICM education standards (2021) and competencies (2024), 15 stakeholder interviews, 3 focus group discussions, and site visits to 4 hospitals and one midwifery school. Quantitative data were mapped using a 4‐point alignment scale, and qualitative data were coded and thematically analyzed.

**Results:**

The Nepal Nursing Council standards for the bachelor's in midwifery program aligned with 72% of the ICM education standards. The standards for the certificate in midwifery program covered 67%, whereas the certificate in midwifery curriculum from the Council for Technical Education and Vocational Training aligned with only 52%. The bachelor's in midwifery curriculum met 89% of the ICM essential competencies, the certificate in midwifery curriculum met 76%, and the certificate in nursing curriculum met 43%. These quantitative gaps were contextualized by qualitative findings, including theory‐practice mismatches, shortages of qualified faculty members and training resources, and insufficient supervision.

**Discussion:**

Stakeholders confirmed the absence of dedicated midwifery regulations and clear career pathways in the country as an issue. Without targeted reforms to standardize curricula, investment in infrastructure, and legally recognizing midwifery as a distinct profession, Nepal's ability to reduce preventable maternal and neonatal mortality will be compromised.

## INTRODUCTION

Midwives, as legally licensed health care professionals trained through internationally recognized programs, play a pivotal role in providing competent and evidence‐based maternal and newborn care. They ensure safe pregnancy, childbirth, and postpartum experiences while promoting women's autonomy and respectful, woman‐centered support.^1^, ^2^

**QUICK POINTS**

✦ This mixed‐methods assessment revealed that Nepal's midwifery education programs are only partially aligned with the International Confederation of Midwives’ (ICM's) global standards and essential competencies.
✦ Although the bachelor's in midwifery curriculum shows high alignment (89%) with ICM competencies, the certificate in nursing curriculum, the most common pathway for maternal‐newborn care providers, covers less than half (43%).
✦ Critical gaps were identified in faculty development as well as theory‐practice mismatch in clinical settings and the absence of a distinct legal and professional framework for midwifery.
✦ Strengthening Nepal's midwifery workforce to improve maternal‐newborn health care outcomes requires targeted policy reforms, including mandated curricular updates, investment in educational infrastructure, and legal recognition of midwifery as an autonomous profession.

Despite progress in reducing maternal, neonatal, and perinatal mortality, Nepal faces plateauing trends, with a maternal mortality ratio of 151 per 100,000 live births, a newborn mortality rate of 21 per 1000 live births, and a stillbirth rate of 10 per 1000 pregnancies at 28 weeks or more.^3^ With an estimated 609,790 live births annually,^4^ maternal and newborn health care challenges remain significant. Although institutional births have increased remarkably, the 2022 survey reports that 19% of births still occur at home, particularly in rural areas and in Madhesh Province, where home births reach 32%.^5^ Improving maternal and newborn outcomes requires strengthened intrapartum care.^6^ Midwives have historically been instrumental in reducing maternal and neonatal mortality and morbidity globally.^7^ When equipped with rigorous education, comprehensive training, and sustained professional support, they can elevate care standards and ensure safer pregnancies and births. Their work also advances global efforts to achieve Sustainable Development Goal 3. Thus, strengthening midwifery education and practice is essential for developing equitable and resilient health care systems to improve maternal and newborn health in the country.

### Nursing and Midwifery Education in Nepal

The Ministry of Health and Population in Nepal introduced a nursing program in 1956, which was managed by Tribhuvan University after its establishment in 1972. Currently, universities and academies oversee bachelor's and master's programs in nursing and midwifery, whereas the Council of Technical Education and Vocational Training (CTEVT) manages the Proficiency Certificate Level (PCL) in Nursing and PCL in Midwifery, a 3‐year diploma after 10 years of schooling. Advanced nursing programs offered at the bachelor's, master's, and doctorate levels focus on developing specialized nursing and midwifery skills.

Despite the unique role of midwives, midwifery is a relatively new profession in Nepal. It was first conceptualized in the 2006 Skilled Birth Attendance policy and formally introduced in 2016.^8^, ^9^ Kathmandu University and the National Academy of Medical Sciences launched bachelor‐level midwifery programs in 2016 and 2017, respectively. Currently, 6 institutions offer bachelor's degrees in midwifery, enrolling approximately 120 students annually. Additionally, CTEVT has managed 2 midwifery schools since 2023, offering a certificate in midwifery, with an annual intake of 60 students. The Nepal Nursing Council (NNC) regulates these programs and sets national standards.^10^ As of May 2025, 91 midwives have been registered and licensed with the NNC. This highlights a critical implementation gap.

A health care system that formally integrates midwives enhances maternal outcomes by assigning the management of physiologic childbirth to their expertise, thereby optimizing the role of obstetricians for high‐risk and pathological cases.^11^ To address these needs, the Ministry of Health and Population's National Human Resource for Health Strategy and Action Plan (2020‐2030) projected the need for 6000 midwives and 410 midwifery officers and specialists by 2030.^12^ In the absence of professional midwives, their roles have traditionally been filled by staff nurses and auxiliary nurse midwives, who undergo an additional 60‐day in‐service training. Although these providers are essential in expanding access, particularly in rural facilities,^13^ their training does not fully equip them to manage complex perinatal cases independently.^14^ Moreover, medical dominance in the health care system limits integration of midwifery as a separate discipline.^15^

The overarching goal of initiating midwifery education in Nepal is to produce highly qualified midwives equipped with midwifery competencies to provide woman‐centered care. However, concerns remain regarding whether current preservice midwifery education adequately prepares graduates to support normal physiologic births and provide respectful, high‐quality care. This highlights the importance of assessing existing curricula against the International Confederation of Midwives (ICM) education standards and midwifery essential competencies.^16^, ^17^ International accreditation efforts underscore the value of standard alignment.^18^ Therefore, this study aimed to provide a comprehensive assessment of Nepal's preservice midwifery education in relation to the ICM standards and midwifery essential competencies. This assessment is crucial for guiding targeted reforms and improvements by identifying gaps in midwifery education standards and critical competencies that will strengthen midwifery education and mobilization, thereby enhancing the quality of maternal and neonatal health care outcomes in Nepal.

## METHODS

### Study Design

This study employed a mixed‐methods approach to comprehensively assess preservice midwifery education in Nepal. The design integrated a quantitative desk review with qualitative data from stakeholder interviews, focus group discussions (FGDs), and on‐site observations. This methodology enabled a systematic examination of midwifery curricula and standards in relation to the ICM global education standards (2021) and midwifery essential competencies (2024) while also capturing the contextual perspectives and lived experiences of key stakeholders. This mixed‐methods study was reported in accordance with the Strengthening the Reporting of Observational Studies in Epidemiology statement and checklist for cross‐sectional studies (Supporting Information: Appendix S1).

### Data Collection

#### Desk Review

The research team conducted a desk review of available national standards, curricula, and documents against the ICM standards for midwifery education and essential competencies, following receipt of ethical approval from the Nepal Health Research Council on July 31, 2022. The primary desk review was conducted between December 2022 and January 2024. Two authors independently reviewed the documents. Discrepancies between reviewers were resolved through discussion with a third reviewer, and information was extracted from the standards set by the NNC, various midwifery curricula, and national policy documents (Supporting Information: Appendix S2). This study mapped the curricula developed between 2012 and 2016 because they are currently implemented against the current global standards updated in 2021 and 2024. This allowed identification of alignment gaps.

#### Stakeholder Interviews and FGDs

Fifteen stakeholder interviews and 3 FGDs were conducted from January 15, 2024, to June 23, 2025, to collect in‐depth perspectives on midwifery education. Participants were selected through purposive sampling to capture diverse perspectives from the policy, education, and practice sectors. These included government officials, educators, health care managers, practicing nurses, auxiliary nurse midwives involved in maternity and newborn care, and current midwifery students (Table 1). Semistructured interview and FGD guidelines (Supporting Information: Appendix S3) were developed based on the study objectives and preliminary findings from the desk review. FGD guidelines were focused on education and practice challenges for midwives.

**Table 1:** Interviews, Focus Group Discussions, and On‐Site Observation Participants and Settings

Data Source	Participant Category	n	Details (Institution Type, Region/Program)
Interviews	Government officials	5	Federal‐ (Ministry of Health) and provincial‐level (Gandaki Province) policymakers
	Health care managers	5	Medical superintendents, matrons, obstetrician‐gynecologists, pediatricians from regional and provincial hospitals
	Regulatory/academic/partner representatives	5	Representatives from NNC, Medical Education Commission, CTEVT, development partners, and the professional midwifery association
FGDs	Teachers from midwifery schools	5	Educators from bachelor's‐ and certificate‐level programs (Gandaki Province)
	Current midwifery students	10	Students from bachelor's‐ and certificate‐level programs (Gandaki Province)
	Practicing nurses/ANMs	5	Maternal and newborn care providers (ANC, PNC, maternity, NICU) from study hospitals
On‐site observations	Hospitals	4	Western regional, Dhaulagiri, Lamjung, and Matrishisu hospitals (regional, provincial, and maternity specialty). Assessed clinical learning environments, resource availability (eg, Doppler monitors), infrastructure, and student supervision
	Midwifery school	1	Gaur technical school (CTEVT). Assessed simulation labs, library resources, and general educational infrastructure

Abbreviations: ANC, antenatal care; ANM, auxiliary nurse‐midwife; CTEVT, Council of Technical Education and Vocational Training; FGD, focus group discussion; NICU, newborn intensive care unit; NNC, Nepal Nursing Council; PNC, postnatal care.

#### On‐Site Visits

On‐site visits were conducted at 4 hospitals and one technical school to observe clinical practice, simulation training, and educational environments. A structured checklist was developed, derived from the ICM Global Standards for Midwifery Education (Supporting Information: Appendix S4) and specifically aligning with category 5 of the ICM standards. A checklist was used to assess the availability and functionality of learning resources, clinical learning environment, supervision and practice conditions, and general infrastructure relevant to midwifery education and service provision. The checklist covered aspects such as infrastructure, equipment availability, student‐teacher ratios in clinical settings, and learning resources, including libraries and laboratories.

### Data Analysis

For the desk review, the midwifery standards and curricula were systematically mapped against the ICM global education standards for midwifery education (2021) and essential competencies (2024). The comparison focused on governance, faculty, students, curriculum, resources, and quality improvements. A detailed table was created to compile findings from these documents.

Each standard or competency, defined as an item in the tables, was assessed for its presence and comprehensiveness in the Nepalese standards and curricula. A 4‐point alignment scale was used for the assessment. The scale was defined as follows: 3 for complete alignment (all subrequirements and requirements of the item were fully and explicitly mentioned), 2 for substantial alignment (>50% of requirements mentioned), 1 for partial alignment (≥1, but ≤50%, of the requirements mentioned), and 0 for no alignment (the item was not mentioned at all). The percentage of alignment for each category was calculated by dividing the number of items scoring 3 (“complete alignment”) by the total number of items in that category and then multiplying the result by 100. Specific areas missing for items with scores of 1 or 2 were noted. The findings are summarized in tabular format (Tables 2 and 3) and validated by sharing them with experts.

**Table 2:** Nepal's National Standards for Midwifery Education Mapped to the ICM Global Standards for Midwifery Education (2021)

		Bachelor's in Midwifery NNC Standards		PCL Midwifery NNC Standards		PCL Midwifery CTEVT	
Standards	Description	Alignment^a^	Gaps	Alignment^a^	Gaps	Alignment^a^	Gaps
1. Program governance	Alignment with jurisdictional policies, governance, leadership roles, and advocacy	5/5 standards (100%)	Limited advocacy roles	4/5 standards (80%)	Limited emphasis on advocacy and external engagement	3/5 standards (60%)	Less defined governance structure; poor emphasis on structured advocacy roles and external engagement
2. Faculty	Midwives, formal teaching preparation, clinical competency, and CPD	6/9 standards (67%)	Faculty CPD and mentorship partially addressed	5/9 standards (56%)	Faculty CPD weak, mentorship roles limited	4/9 standards (44%)	Weakness in provisions for ongoing CPD or formal mentorship for faculty
3. Students	Admission policies, equity, clinical practice exposure, and well‐being	5/10 standards (50%)	Moderate equity considerations	5/10 standards (50%)	Limited equity considerations	5/10 standards (50%)	Limited strategies for ensuring equity in admissions; minimal guidance on student welfare, such as counseling services
4. Midwifery program and curriculum	Integration of ICM competencies, program length, balance of theory (40%) and practice (50%)	9/11 standards (82%)	No specific gaps identified	8/11 standards (73%)	No specific gaps identified	8/11 standards (73%)	The theory and practice balance is well defined; however, the content could be more granularly detailed
5. Resources	Sufficient infrastructure, learning materials, diverse clinical sites, and student support services	5/7 standards (72%)	Comprehensive resource requirements	6/7 standards (86%)	Comprehensive resource guidelines	3/7 standards (43%)	Lacks detailed guidelines for ensuring the quality and adequacy of infrastructure and clinical sites and comprehensive student support services
6. Quality improvement	Internal and external reviews, continuous monitoring, and public reporting of outcomes	3/4 standards (75%)	Internal reviews lack external evaluations and public reporting	2/4 standards (50%)	Internal reviews placed limited emphasis on external reviews	0/4 standards (0%)	It was updated in 2023; however, lacks defined mechanisms for quality improvement
**Total coverage**		**33/46 standards (72%)**		**31/46 standards (67%)**		**24/46 standards (52%)**	

Abbreviations: CPD, Continuous Professional Development; CTEVT, Council of Technical Education and Vocational Training; ICM, International Confederation of Midwives; NNC, Nepal Nursing Council; PCL, Proficiency Certificate Level.

a A 4‐point scale was used: 3 (all requirements of the items were fully and properly mentioned/complete alignment), 2 (>50% of requirements mentioned/substantial alignment), 1 (≥1, but ≤50%, of the requirements mentioned/partial alignment), and 0 (none of the requirements mentioned/no alignment). The percentage of alignment for each category was calculated by dividing the number of items scoring 3 “complete alignment” by the total number of items in that category and multiplying it by 100.

**Table 3:** Nepal's Midwifery Curricula Mapped to the ICM Essential Competencies for Midwifery Practice (2024)

		Bachelor's in Midwifery		PCL Midwifery		PCL Nursing	
Essential Competencies for Midwifery	Description of Coverage Areas	Alignment^a^	Gaps	Alignment^a^	Gaps	Alignment^a^	Gaps
Cross‐functional competencies for midwifery practice	Autonomy and accountability, continuing education, delegation and supervision, research to inform practice, adherence to laws and codes of conduct, human rights in midwifery care, support for women's choices, effective communication, collaboration with health care professionals, health care, health assessment and promotion, prevention and treatment of common health problems, recognition and management of complications, facilitation of normal birth processes, and prescription and administration of medicines	14/16 competencies (85%)	Adoption of modern technologies, care in humanitarian crises Lack of training in legal frameworks for autonomous practice, burnout prevention, telehealth, and AI‐driven diagnostics Emergency preparation is limited. Advocacy for marginalized groups, cultural competency, and interprofessional simulations are underrepresented, whereas protocols for adolescent health screening, anemia, urinary tract infections, disaster preparedness, and alternative birthing positions are absent.	11/16 competencies (69%)	Continuing education programs, training in modern technologies, delegation practices, training for humanitarian crises Advanced research competencies Advanced management techniques for specific obstetric emergencies or leadership roles	4/16 competencies (25%)	Inadequate content in personal safety, self‐care, continuing education, and adaptation to emerging health care technologies. There is no coverage of legal frameworks for delegation and oversight, limiting nurse midwives’ ability to supervise and mentor others. Additionally, the curriculum does not address midwifery competencies in humanitarian crises, including reproductive health care needs in disaster settings and climate‐related impacts. Obstetric referral competencies
Sexual and reproductive health care and rights	Education on sexual and reproductive health care, support for natural family planning and barrier methods, administration of contraceptives, and preconception care	4/6 competencies (67%)	Care for victims of violence and abuse and comprehensive abortion care Gaps include training on natural family planning, subdermal implants, trauma‐informed care, forensic evidence collection, and postabortion mental health.	3/6 competencies (50%)	Preconception care, care for victims of violence, comprehensive abortion care	2/6 competencies (33%)	Gaps exist in preconception care. Training on caring for victims of violence and abuse is insufficient, with no coverage of WHO guidelines, signs of abuse, or referral mechanisms. Comprehensive abortion care is also not covered, including legal frameworks, safe abortion methods, postabortion care, and emergency protocols.
Antenatal care	Health status and pregnancy assessment, fetal well‐being assessment, monitoring pregnancy progression, promotion of health behaviors, anticipatory guidance, management of complicated pregnancies, and birth planning	7/7 competencies (100%)	Lacks education on cardiotocography interpretation, Doppler ultrasound, substance abuse counseling, parenting education, and protocols for gestational diabetes, placenta accreta, and home birth preparedness	7/7 competencies (100%)	No specific gaps identified	5/7 competencies (71%)	Inadequate contents in health status assessment, fetal monitoring, pregnancy progression, health care promotion, anticipatory guidance, complicated pregnancy management, and birth planning
Care during labor and birth	Promotion of normal labor and birth, management of safe vaginal birth, and newborn care immediately after birth	3/3 competencies (100%)	Training is deficient in waterbirth, upright birthing positions, advanced perineal repair, and preterm stabilization.	2/3 competencies (67%)	No specific gaps identified	2/3 competencies (67%)	Encompassing physiologic labor promotion, safe vaginal birth, complication prevention, and immediate newborn care Use of the Labor Care Guide
Ongoing care of women and newborns	Postnatal care for women who are healthy, care for newborns who are healthy, promotion and support of breastfeeding, detection and management of postnatal complications, and detection and management of newborn health problems	5/5 competencies (100%)	Lacks validated depression screening, newborn metabolic screening, relactation, donor milk use, postpartum psychosis, venous thromboembolism, neonatal sepsis, and jaundice phototherapy	5/5 competencies (100%)	No specific gaps identified	3/5 competencies (60%)	Gaps in covering postnatal care, newborn care, breastfeeding support, and the detection and management of postnatal complications Newborn resuscitation
**Total coverage**		**33/37 competencies (89%)**		**28/37 competencies (76%)**		**16/37 competencies (43%)**	

Abbreviations: Al, artificial intelligence; ICM, International Confederation of Midwives; PCL, Proficiency Certificate Level; WHO, World Health Organization.

a A 4‐point scale was used: 3 (all requirements of the items were fully and properly mentioned/complete alignment), 2 (>50% requirements mentioned/substantial alignment), 1 (≥1, but ≤50%, of the requirements mentioned/partial alignment), and 0 (none of the requirements mentioned/no alignment). The percentage of alignment for each category was calculated by dividing the number of items scoring 3 “complete alignment” by the total number of items in that category and multiplying it by 100.

Interviews and FGDs were audio‐recorded with informed consent, transcribed verbatim, and translated into English by the first author. The transcripts were thematically analyzed following the approach described by Braun and Clarke,^19^ integrating both inductive and deductive (ICM framework‐guided) coding. Two authors independently coded the transcripts for reliability, and discrepancies were resolved by a third coder. The team discussed the codebook to finalize themes. Findings from the on‐site visits were recorded in an Excel sheet and analyzed thematically to identify recurring observations regarding facilities, resources, and practice environments. Themes were developed iteratively, and analysis was supported by NVivo 15 qualitative data analysis software.^20^

### Ethical Approval

Ethical approval was obtained from the Nepal Health Research Council (number 253—2022) and the Ethics Commission of Ludwig‐Maximilians‐Universität (number 23—0488). Written informed consent was obtained from all interview and FGD participants before data collection. Participation was voluntary, and participants were assured of confidentiality and anonymity. Data were stored securely and accessed only by the research team.

### Positionality and Reflexivity

The research team included Nepali public health care researchers, midwives, and international experts from Ludwig‐Maximilians‐Universität, providing both local and global perspectives on the issue. Data collection was conducted by trained Nepali researchers in the local language, and data collectors minimized bias throughout the process. The team held regular debriefings to discuss themes and challenge assumptions.

## RESULTS

The findings from the assessment are presented below, detailing the alignment of Nepalese midwifery education standards and curricula with ICM global education standards and essential midwifery competencies, followed by key themes from stakeholder interviews, FGDs, and on‐site visits.

### Comparison of Bachelor's in Midwifery and Certificate in Midwifery Standards in Relation to ICM Standards for Midwifery Practice

A comparative desk review of the bachelor's and certificate in midwifery standards revealed substantial but incomplete alignment with the ICM education standards (Table 2). The bachelor's in midwifery program demonstrated the highest level of adherence, whereas the certificate programs showed moderate to limited alignment, particularly in areas such as faculty development, student support, and quality assurance. Overall, the findings highlight persistent gaps in governance, resources, and continuous professional development, underscoring the need for systematic standardization across all preservice midwifery programs.

### Comparison of Bachelor's in Midwifery, Certificate in Midwifery, and Certificate in Nursing Curricula in Relation to ICM Essential Competencies for Midwifery Practice, 2024

The desk review compared the bachelor's in midwifery, certificate in midwifery, and certificate in nursing curricula against the ICM essential competencies (2024), demonstrating clear variation in alignment (Table 3). The bachelor's in midwifery curriculum achieved the strongest conformity, whereas the certificate in midwifery and certificate in nursing curricula showed declines in coverage, particularly across advanced clinical competencies and woman‐centered care. Educational gaps were also noted in modern technologies, leadership, and evidence‐based practice, reflecting broader systemic challenges in curriculum design, faculty preparation, and clinical practices. These disparities indicate that lower‐level programs may insufficiently prepare graduates to meet the full scope of midwifery practice within Nepal's current educational and regulatory environment.

### Stakeholder Perceptions of Midwifery Education Gaps

The qualitative findings from the stakeholder interviews and FGDs complemented and contextualized the gaps identified in the desk review. Participants’ concerns were aligned with gaps in curriculum, distribution, and regulation.

#### Perceptions of Curricular and Clinical Competency Gaps

Most graduates of the certificate in nursing program reported feeling inadequately equipped in midwifery clinical skills, consistent with the finding that this curriculum only met 43% of the ICM's essential competencies. Participants of the certificate in midwifery course also identified a substantial gap between theory and practice. As one midwifery graduate explained, “While the midwifery model of care is emphasized in classroom settings and simulation labs, clinical sites often follow the obstetric model, making it challenging to implement classroom teachings in practice” (FGD‐Graduate‐01).

#### Challenges in Midwifery Education, Delivery, and Implementation

Challenges in midwifery education emerged most prominently in the discussions. Stakeholders strongly endorsed the desk review's conclusions regarding weaknesses in faculty availability, professional development, and learning resources. A primary concern was the shortage of adequately trained midwifery educators and the lack of essential teaching materials, such as reference books. One faculty member noted, “There is a lack of training materials, including a simulation lab” (Interview‐Faculty‐02). Moreover, one certificate of midwifery student added, “We lack adequate books and reference materials to support our learning” (FGD‐Student‐03).

Similar challenges were observed in clinical sites with limited equipment and high workloads: “We often face shortages of essential equipment such as Doppler monitors, and the heavy workload makes learning stressful” (FGD‐Student‐05). A key issue identified was the inadequacy of clinical placements. Students frequently reported poor supervision, overcrowded facilities, and limited access to an adequate number of births, leaving them unprepared for clinical practice: “Sometimes we cannot observe enough births due to overcrowded facilities and lack of supervision” (FGD‐Student‐06). These findings align with the findings from the desk review, which indicated that only 50% of the students’ standard—encompassing clinical exposure—was met in the NNC standards.

#### Challenges in Regulation and Professional Pathways

“Stakeholders also expressed concerns related to professional identity and future career prospects, reflecting systemic gaps in regulations and governance. A recurring theme was the absence of a structured career pathway for midwives, resulting in uncertainty regarding their professional roles after graduation. One student voiced this concern: “What happens after graduation?” I need clear pathways for my career and assurance of job postings” (FGD‐Student‐07).

Stakeholders also mentioned the need for incentives to support retention. The absence of midwifery‐specific legislation and dedicated positions within the health care system was cited as a key barrier. Staff nurses noted, “We were educated as midwives but still work as staff nurses. Since there are no actual midwife positions, we worry about our future” (Interview‐StaffNurse‐01).

Despite stated political support for midwifery, activities to produce midwives remain limited. As one policy maker remarked, “We are committed to starting and advancing midwifery education and practices. This is crucial for the health of our mothers and newborns” (Interview‐Policymaker‐01). However, this commitment has not yet been translated into implementation. A recent graduate expressed this sentiment, stating, “Many of us newly graduated midwives are anxious. There is a lack of clarity regarding where we will be deployed. We need a sharp vision and strategies in place” (FGD‐Graduate‐02). The absence of a robust regulatory framework and a clearly defined professional pathway has limited the effective implementation and advancement of midwifery education.

#### Stakeholder Recommendations for Improvement

The participants also proposed solutions to address these challenges. A key recommendation was to create a defined professional structure supported by graduates, educators, and managers. Policymakers emphasized the need for dedicated midwife positions in the government structures, whereas graduates sought clear career pathways. Participants emphasized the importance of improving faculty capacity, learning resources, and simulation facilities.

## DISCUSSION

This study assessed the alignment of preservice midwifery education in Nepal with the ICM's global standards and essential competencies. Despite gradual progress, significant gaps persist that must be addressed to strengthen the midwifery profession and improve maternal and newborn health care outcomes. The findings are interpreted in relation to existing literature and key thematic areas. Nepal's national curricula, developed between 2012 and 2016, show limited alignment with current ICM global standards from 2021 and 2024. This reveals systemic delays in educational policy and quality assurance because preservice training fails to align with global evidence‐based practice.

### Competency and Curricular Gaps

Our analysis revealed notable variation in the extent to which the curricula were aligned with the ICM essential competencies, with the bachelor's program demonstrating the strongest alignment, followed by the certificate in midwifery and certificate in nursing programs, which showed progressively lower levels of conformity. Qualitative findings provide context for quantitative results.

This incomplete alignment highlights the need for urgent curricular updates,^10^ a finding that resonates with extensive global literature on the inconsistent implementation of ICM standards globally.^15^, ^21^, ^22^ For instance, a 2023 study in Iran identified similar discrepancies between its national curriculum and ICM competencies.^21^ Our findings also support findings from Australia and a 4‐country assessment in Africa (Benin, Malawi, Tanzania, and Uganda), which all identified significant gaps between national training programs and global standards.^15^, ^23^ Similar regulatory weaknesses are reported in other South Asian countries^24^ and the Democratic Republic of Congo.^25^

This need for curricular reform is not limited to technical skills. Deficiencies are also evident in key areas such as perinatal mental health.^26^ Our findings also align with other studies highlighting shortcomings in advanced competencies,^27^ insufficient midwifery knowledge among educators,^28^ and a poor application of curricula.^29^ Furthermore, the gap in woman‐centered, rights‐based care is a well‐documented global issue,^30^, ^31^, ^32^ which our study also confirms as an issue in Nepal, because training on respectful maternity care also remains limited. These deficiencies limit students’ capacity to address complex maternal and newborn health issues, underscoring the need for regular curriculum review and updates.^21^ In the certificate in nursing curriculum, limited midwifery content constrains the ability to provide comprehensive services, making reliance on in‐service training a temporary and cost‐inefficient solution.

Our analysis (Table 3) shows both foundational and contemporary deficiencies in Nepal's midwifery curricula. The recent ICM standards (2021/2024) highlight new competencies, such as telehealth, technology‐driven diagnostics, care in humanitarian crises, and legal frameworks for autonomous practice, that were not included when the current curricula were developed. Even the bachelor's curriculum, which showed the highest level of alignment, showed shortcomings in these areas, whereas the certificate in nursing program was notably deficient in key components of rights‐based care, including the management of violence and comprehensive abortion care. This reflects a dual challenge of current curricula and structural gaps. The curricula has gaps in relation to evolving global standards, whereas persistent systemic weaknesses, such as limited practical exposure, inadequate supervision, and a shortage of trained educators, continue to impede the achievement of competencies. Overall, Nepal's midwifery education system confronts both enduring foundational gaps and a delay in adapting to the expanding global scope of midwifery practice.

### Midwifery Education Delivery and Implementation Gaps

Beyond the documented curricular content, significant challenges persist in providing midwifery education. Qualitative findings suggest that graduates perceive themselves as inadequately skilled, indicating that limited clinical exposure hinders their ability to apply theoretical knowledge in practice. This was particularly evident among certificate graduates, who felt poorly equipped because of insufficient supervision and limited case exposure.^33^ These deficiencies hinder competency and mirror findings from other settings.^28^, ^34^, ^35^, ^36^

These implementation challenges are compounded by a shortage of qualified educators, inadequate infrastructure, insufficient training materials, and poorly equipped clinical sites. These issues align with findings from other studies, which report a lack of formal midwifery qualifications among educators, weak regulatory oversight, and a need for structured faculty development.^37^, ^38^ Improving educator capacity is essential. Resource disparities and poor infrastructure further limit effective learning, ultimately contributing to the production of graduates who may lack the competencies to provide quality care.^35^, ^39^

### Accreditation, Regulation, and Professional Support Gaps

Midwifery in Nepal still essentially functions as a component of nursing, lacking distinct recognition as an independent profession. Nepal lacks specific national legislation for midwifery, and the profession is regulated by the NNC. This contradicts the ICM recommendations. The ICM defines an autonomous profession through its unique knowledge, self‐governance, and societal recognition, achieved through regulation.^40^ Qualitative findings validated stakeholder concerns regarding role ambiguity, reinforcing the literature that highlights weak legal recognition and the need for a more distinct professional identity.^15^ This reflects persistent medical dominance. In the United States, midwives face state‐specific practice limitations that require physician supervision, paralleling the barriers to autonomous practice in our findings.^41^, ^42^ These barriers at the practice level undermine educational progress in the absence of proper legal frameworks. Lack of independent regulation limits professional identity and quality.^15^, ^24^

Moreover, the quality assurance mechanisms are weak. A deficiency in systematic quality improvement initiatives, accreditation, and ongoing monitoring aligned with ICM standards is a significant concern affecting educational consistency and quality.^43^ The literature emphasizes that continuous quality improvement is essential^37^, ^44^ and that inadequate infrastructure and educator deficiencies fundamentally undermine quality.^35^ Finally, qualitative findings revealed challenging work environments, including resource constraints and understaffing, as well as uncertain career paths and deployment concerns among students. These issues are consistent with challenges in other low‐income and middle‐income countries,^45^ where reports also highlight a disconnect between the production of graduates and the availability of formal deployment pathways,^46^ risking undermining efforts to strengthen the workforce by contributing to stress, burnout, and attrition among service providers. Although interest in midwifery is strong, concerns about job security and career growth limit the profession. Addressing these challenges requires collaboration among stakeholders, clinical training, and effective deployment strategies.

### Policy Implications of Divergent Educational Pathways

The competency gaps between the bachelor's in midwifery and the certificate in midwifery programs present a key policy challenge. Although a bachelor's degree meets global standards,^40^ certificate‐level cadres remain vital for maternal care in remote regions. Discontinuing the PCL pathway reduces access to care. Similar training gaps are seen across South Asia.^47^ The solution is to create a tiered system by improving PCL curricula to meet basic competencies, define practice limits, and develop “bridge” programs for advancement. This strategy for midwifery models^48^ requires strong leadership^46^ for feasible implementation. New Zealand and the Netherlands have established autonomous midwifery practice through legislation, university education, and protected professional pathways.^49^, ^50^ These frameworks can inform Nepal's Midwifery Roadmap.

### Limitations

This study had several limitations that should be considered. The desk review relied on documented standards and curricula, which may not fully reflect the actual provision of teaching and learning practices in the field. To address this issue, we employed a triangulation method that integrated desk reviews with observations and interviews. This approach confirmed that the documented deficiencies in resources and faculty are indeed present in practice. Although the qualitative data were rich, they were collected from a specified set of stakeholders and sites, which may not be fully representative of the entire country. However, we sought to mitigate this by using purposive sampling to include a wide range of key stakeholders in policy, education, and clinical practice. The cross‐sectional design provides a snapshot of time and cannot capture changes over time.

### Implications and Recommendations

This study highlights the need for reforms in curriculum design, implementation, and regulation to strengthen midwifery education and practice in Nepal. Aligning with ICM global standards requires a systematic approach to curriculum development, monitoring, and evaluation.

#### Curriculum Reform

Regular midwifery curriculum reviews are crucial for maintaining alignment with ICM competencies and avoiding outdated content. Evaluative research should assess the effects of curriculum changes on graduate competency and maternal‐newborn health care outcomes. The NNC and universities should institutionalize 5‐year curriculum reviews, consistent with those of other health care professions. The ICM toolkits can be used to guide curriculum updates.

#### Strengthening the Delivery and Implementation of Midwifery Education

Bridging the theory‐practice gap requires faculty capacity building and improved training infrastructure. Faculty development should emphasize pedagogical and simulation‐based teaching, and modular learning models can help mitigate staff shortages. Clinical training should be strengthened through extended placements and simulation laboratories.

#### Professional and Regulatory Advancement

Advancing midwifery in Nepal requires legal recognition as an autonomous profession, distinct from nursing, to ensure clarity in educational standards and the scope of practice. Establishing defined career pathways, deployment strategies, and supportive work environments with adequate resources and staffing ratios will promote retention and quality of care. Further research should explore how educational and regulatory gaps influence the outcomes of services. The findings are being shared with the Ministry of Health and Population, NNC, and partners to inform the development of the forthcoming Midwifery Roadmap.

## CONCLUSION

Despite measurable progress, midwifery education in Nepal remains insufficiently aligned with international standards, particularly those set by the ICM. This study identifies key structural deficiencies in curriculum content, instructional quality, and regulatory recognition, which collectively undermine the development of a competent midwifery workforce. Curricula lack comprehensive integration of essential competencies within the certificate in nursing pathway, revealing significant gaps in cross‐functional competencies and sexual and reproductive health care rights. Educational distribution is constrained by limited faculty expertise and insufficient training resources. Furthermore, the absence of formal legal autonomy restricts the professional identity and scope of midwifery in the national health care system. Reforms are needed to address these issues. Prioritizing evidence‐informed curriculum updates, strengthening institutional capacity, and enacting legal provisions to define midwifery as a distinct profession are crucial steps. These measures will enhance workforce capability and contribute to reducing maternal and neonatal morbidity and mortality, thereby supporting Nepal's commitment to achieving national and global health care goals.

## CONFLICT OF INTEREST

The authors have declared that no conflict of interest exists.

## Supporting information




**Appendix S1**. Strengthening the Reporting of Observational Studies in Epidemiology (STROBE) checklist

**Appendix S2**. Global and National Midwifery Standards, Curricula, and Policy Frameworks

**Appendix S3**. Interview and Focus Group Discussion Guide

**Appendix S4**. Observation Checklist
