# Supplementary material for: Alignment of Midwifery Education in Nepal With Global Standards and Essential Competencies From the International Confederation of Midwives: A Mixed‐Methods Study
Source: J Midwifery Womens Health. 2026 Apr 6;71(3):367–78. doi: 10.1111/jmwh.70096 (PMC13263916; doi:10.1111/jmwh.70096)
Supplement: Supplementary file 1 — Appendix S1. Strengthening the Reporting of Observational Studies in Epidemiology (STROBE) checklist [file JMWH-71-367-s003.docx]

### Supplementary File 1: STROBE Checklist

**STROBE Statement**: Checklist of items that should be included in reports of cross-sectional studies for the manuscript titled, "Assessing Midwifery Education in Nepal with Global Standards and Essential Competencies: A Mixed-Methods Study."

| **Item No.** | **Recommendation** | **Reported on Page # (or Section/Source #)** |
| --- | --- | --- |
| **Title and abstract** |  |  |
| 1 | (a) Indicate the study’s design with a commonly used term in the title or the abstract. | **Yes**. Title and Abstract |
|  | (b) Provide an informative and balanced summary of what was done and what was found in the abstract. | **Yes**. Abstract |
| **Introduction** |  |  |
| 2 | **Background/rationale**—Explain the scientific background and rationale for the investigation being reported. | **Yes**. Introduction |
| 3 | **Objectives**—State specific objectives, including any prespecified hypotheses. | **Yes**. Introduction |
| **Methods** |  |  |
| 4 | **Study design**—Present key elements of the study design early in the paper. | **Yes**. Methods, Study design |
| 5 | **Setting**—Describe the setting, locations, and relevant dates, including periods of recruitment, exposure, follow-up, and data collection. | **Yes**. The Methods section specifies N) and the setting, details the data collection period (January 15, 2024–June 23, 2025), and lists the specific hospitals and schools visited. |
| 6 | **Participants**—(a) Give the eligibility criteria, and the sources and methods of selection of participants. | **Yes**. Participants were selected via purposive sampling. Eligibility is described by professional role (e.g., government officials, educators, students, etc.) in the Methods section and Table 1. |
| 7 | **Variables**—Clearly define all outcomes, exposures, predictors, potential confounders, and effect modifiers. Give diagnostic criteria, if applicable. | **Yes**. The primary outcome, the alignment of curricula/standards with ICM standards, is clearly defined using a 4-point scale. Qualitative themes were also represented. |
| 8 | **Data sources/ measurement**—For each variable of interest, give sources of data and details of methods of assessment (measurement). | **Yes**. Data sources (national curricula, policy documents, interviews, FGDs, site visits) and measurement tools (4-point alignment scale, semi-structured interview guides, structured checklist) are described in the Methods section. |
| 9 | **Bias**—Describe any efforts to address potential sources of bias. | **Yes**. Addressed potential reviewer bias in the desk review through independent review by two authors and reconciliation by a third. Acknowledged the temporal mismatch of documents as a limitation. |
| 10 | **Study size**—Explain how the study size was arrived at. | **Yes**. The number of documents, interviews (15), FGDs (3), and participants (35 total) is mentioned, and data saturation was reached. |
| 11 | **Quantitative variables**—Explain how quantitative variables were handled in the analyses. Describe which groupings were chosen and why. | **Yes**. The data analysis section explains that a 4-point scale was used to calculate the percentage of compliance for each pre-defined category based on the ICM standards. |
| 12 | **Statistical methods**—(a) Describe all statistical techniques, including those used to control for confounding. | **Yes**. The manuscript describes the descriptive quantitative method (calculation of alignment percentages) and the qualitative method (thematic analysis using NVivo). |
| **Results** |  |  |
| 13 | **Participants**—(a) Report the number of individuals at each stage of the study. | **Yes**. Reports 15 stakeholder interviews and 3 FGDs with a total of 35 participants. The number of documents and sites visited is also reported. |
| 14 | **Descriptive data**—(a) Give characteristics of study participants (e.g., demographic, clinical, social) and information on exposures and potential confounders. | **Yes**. Table 1 provides the professional roles and the number of participants in each category. |
| 15 | **Outcome data**—Report the main quantitative results (alignment percentages) for each program. | **Yes**. Alignment percentages are reported in the Results section text and summarised in Tables 2 and 3. |
| 16 | **Main results**—(a) Present key results from the desk review and qualitative analysis. | **Yes**. The Results section presents the quantitative alignment data and the main qualitative themes, supported by participant quotes. |
| 17 | **Other analyses**—Report other analyses done—e.g., analyses of subgroups and interactions, and sensitivity analyses. | **Yes**. The entire study is a subgroup analysis, comparing the alignment of different midwifery and nursing programs (Bachelor vs. PCL Midwifery vs. PCL Nursing). This is the primary analysis. |
| **Discussion** |  |  |
| 18 | **Key results**—Summarise key results regarding study objectives. | **Yes**. The Discussion section begins by summarising the key findings, such as the variation in curricular alignment across different programs. |
| 19 | **Limitations**—Discuss limitations of the study, taking into account sources of potential bias or imprecision. Discuss both the direction and magnitude of any possible bias. | **Yes**. A dedicated "Limitations" section is provided, discussing reliance on documented curricula, potential lack of representativeness of qualitative data, and the cross-sectional design. |
| 20 | **Interpretation**—Give a cautious overall interpretation of results considering objectives, limitations, multiplicity of analyses, results from similar studies, and other relevant evidence. | **Yes**. The Discussion interprets findings in the context of existing literature from other countries and discusses the practical implications of the identified gaps. |
| 21 | **Generalisability**—Discuss the generalisability (external validity) of the study results. | **Yes**. The Limitations section explicitly notes that findings may not be representative of the entire country. The Discussion also notes that the challenges identified are consistent with those in other low- and middle-income countries. |
| **Other information** |  |  |
| 22 | **Funding**—Give the source of funding and the role of the funders for the present study and, if applicable, for the original research on which the present article is based. | **Yes.** Reported in the 'Statements & Declarations' section of the Title Page. |
