# Supplementary material for: Alignment of Midwifery Education in Nepal With Global Standards and Essential Competencies From the International Confederation of Midwives: A Mixed‐Methods Study
Source: J Midwifery Womens Health. 2026 Apr 6;71(3):367–78. doi: 10.1111/jmwh.70096 (PMC13263916; doi:10.1111/jmwh.70096)
Supplement: Supplementary file 2 — Appendix S2. Global and National Midwifery Standards, Curricula, and Policy Frameworks [file JMWH-71-367-s002.docx]

**Appendix S2. Global and National Midwifery Standards, Curricula, and Policy Frameworks**

The following table provides the titles and, where available, the persistent uniform resource locators (URLs) for the documents cited in the method. These documents delineate the transition from the Skilled Birth Attendant (SBA) model to the professionalization of midwifery in Nepal.

| **Category** | **Document Title** | **Digital Access / Source** |
| --- | --- | --- |
| **ICM Standards** | ICM Global Standards for Midwifery Education (2021) | <https://internationalmidwives.org/wp-content/uploads/ICM-GLOBAL-STANDARDS-FOR-MIDWIFERY-EDUCATION-1.pdf> |
|  | ICM Global Standards of Essential Competencies (2024) | <https://internationalmidwives.org/resources/essential-competencies-for-midwifery-practice/> |
| **National Standards** | NNC Minimum Requirements for PCL Midwifery Program (2018) | <http://www.nnc.org.np/pages/download/minimum-requirements.php> |
|  | NNC Minimum Requirements for PCL Nursing (2016) | <http://www.nnc.org.np/pages/download/minimum-requirements.php> |
|  | NNC Minimum Requirements for Bachelor's in Midwifery (2016) | <http://www.nnc.org.np/pages/download/minimum-requirements.php> |
| **Pre-service Curricula** | PCL Midwifery Curriculum (2024) | <https://ctevt.org.np/uploads/docs/2024-02-27_pcl_midwifery_updated_2023.pdf> |
|  | PCL Nursing Curriculum (2018) | <https://ctevt.org.np/uploads/docs/2020-05-11_PCL%20Nursing,%202013.pdf> |
|  | Bachelor Level Midwifery Curricula from various universities and academies | Available upon request from corresponding author |
| **Policy Documents** | Skilled Birth Attendant (SBA) Policy (2006) | <https://fwd.gov.np/category/publications> |
|  | National Health Policy (2019) | <https://mohp.gov.np/content/162/national-health-policy-2071/> |
|  | Safe Motherhood and Newborn Health Road Map 2019–2030 | <https://fwd.gov.np/site_uploads/2021/08/SMNH-Roadmap-2030-SGOP.pdf> |
|  | National Human Resources for Health Strategy 2021–2030 | <https://mohp.gov.np> or  <https://publichealthupdate.com/hrhstrategy2030nepal/> |
|  | National Nursing and Midwifery Strategy 2021–2030 | <http://nssd.dohs.gov.np/downloads/Nursing_And_Midwifery_Strategies_2022.pdf> |
|  | National Midwifery Roadmap 2025–2035 | <http://nssd.dohs.gov.np> or Available upon request from corresponding author |
