# Supplementary material for: Alignment of Midwifery Education in Nepal With Global Standards and Essential Competencies From the International Confederation of Midwives: A Mixed‐Methods Study
Source: J Midwifery Womens Health. 2026 Apr 6;71(3):367–78. doi: 10.1111/jmwh.70096 (PMC13263916; doi:10.1111/jmwh.70096)
Supplement: Supplementary file 3 — Appendix S3. Interview and Focus Group Discussion Guide [file JMWH-71-367-s001.pdf]

Key Informant Interview Guideline

Target Participants: Teachers, Faculty Heads, and Principals of Midwifery Schools in Nepal.

Objective: To gather in-depth, expert perspectives from midwifery educators and institutional leaders on the challenges, gaps, and opportunities within midwifery education in Nepal. This will add qualitative depth to the study's preliminary findings regarding curriculum, faculty development, resources, and clinical training.

Time Allotment: Approximately 45-60 minutes per interview.

Interviewer's Opening Script

"Good morning/afternoon and thank you for your time. My name is [Interviewer's Name], and I am part of the research team assessing midwifery education in Nepal. Our study has reviewed the national curricula and standards, and we have some preliminary findings. A crucial part of our work now is to speak with experienced educators and leaders like you to understand the context behind these findings and to hear your expert opinions.

The interview will take about 45-60 minutes. As we discussed in the consent form, this conversation is confidential, and your name will not be used in any reports. With your permission, I will be audio-recording our conversation to ensure I capture your valuable insights accurately. Do you have any questions before we begin?"

Interview Questions by Theme

Theme 1: Professional Background and Role (5 mins)

- Could you please start by briefly describing your role at this institution and your experience in the field of midwifery or nursing education?
- What do you see as the primary mission of your midwifery program?

Theme 2: Curriculum and Teaching Practices (15 mins)

- Our study found that while the Bachelor of Midwifery (BMS) curriculum aligns well with international competencies, gaps exist, particularly in the PCL programs. From your perspective, how well does the curriculum you teach prepare students for the realities of midwifery practice in Nepal?
- A teacher in our study noted a gap between the "midwifery model of care" taught in classrooms and the "obstetric model" students often see in clinical sites. Have you observed this? How do you and your faculty address this discrepancy in your teaching?
- What are the biggest challenges you face in delivering the curriculum as it is designed?

Theme 3: Faculty Development and Support (10 mins)

- The preliminary findings highlight a need for more robust faculty development, including mentorship and Continuous Professional Development (CPD). What opportunities for professional growth and training are currently available to you and your staff?
- A significant challenge identified was the shortage of specialized and formally qualified midwifery educators. How does this affect your institution, and what solutions do you think are most practical to address this?

Theme 4: Resources, Infrastructure, and Quality Assurance (10 mins)

- We've heard from stakeholders about challenges with inadequate teaching resources, such as a "lack of training materials including simulation lab". Could you describe the state of teaching facilities, equipment, and learning resources at your school?
- Our review of the standards showed that mechanisms for quality improvement, such as external reviews and public reporting, are sometimes weak or absent. What processes does your institution have in place to monitor and improve the quality of the midwifery program?

Theme 5: Clinical Practice and Supervision (10 mins)

- A recurring theme is the challenge of ensuring adequate clinical exposure and supervision for students. From your position, what are the main barriers to providing high-quality clinical placements?
- How does the school collaborate with hospitals and clinical sites to ensure students meet their learning objectives? How are clinical preceptors or supervisors selected and supported?

Interviewer's Closing Script

"Thank you very much for sharing your detailed and insightful perspectives. This has been incredibly helpful for our research. The information you've provided will contribute significantly to developing meaningful recommendations for strengthening midwifery education in Nepal. Do you have any final thoughts or questions for me?"

Focus Group Discussion Guideline: Exploring Midwifery Students' Perspectives

Objective: To gather in-depth qualitative insights from midwifery students to explore, contextualize, and add depth to the preliminary quantitative findings of an ongoing assessment of midwifery education in Nepal.

Participants: Current students enrolled in Bachelor of Midwifery Sciences (BMS) or Proficiency Certificate Level (PCL) Midwifery programs.

Time Allotment: 60-75 minutes

Materials:

- Consent forms
- Audio recorder and notepad for note-taking
- Quiet, private room
- Refreshment

---

1. Introduction & Warm-Up (10 minutes)

(Facilitator's Script)

"Good morning/afternoon and thank you for joining us today. My name is [Facilitator's Name], and I am part of a research team. We are currently conducting a study to assess midwifery education in Nepal. As part of our initial work, we have reviewed national standards and curricula and have some preliminary quantitative findings.

A crucial next step in our research is to hear directly from you. Your experiences as students are essential to help us explore these initial findings more deeply and to understand the real-world context behind the numbers. Your participation is voluntary, and everything you share will be kept confidential. We will not use your names in any reports. We are recording the session to ensure we capture all your valuable insights accurately. Please feel free to speak openly and honestly; we are here to learn from your unique perspectives.

Before we begin, do you have any questions? [Address questions]. Have you all signed the consent form? [Confirm consent].

Let's start with a quick round of introductions. Please tell us your name and what inspired you to study midwifery."

---

2. Theme 1: Educational Experience and Curriculum (15-20 minutes)

Objective: To explore students' perceptions of their academic learning in light of the study's preliminary findings on curriculum and resource alignment.

Key Questions:

1. To begin, could you share your general feelings about the quality of the teaching in your program?
2. Our initial analysis of educational standards suggests there can be gaps in resources, with some standards lacking detailed guidelines for infrastructure or student support services. We want to understand what this looks like from a student's point of view. What has been your experience with the learning materials and facilities, like simulation labs or libraries, available to you?
3. Our quantitative review shows that the Bachelor in Midwifery curriculum has a higher alignment with international competencies (89%) compared to the PCL Midwifery (76%) or PCL Nursing (43%) curricula. From your perspective as a student, do you notice a gap between what is taught in the classroom—what we might call the 'midwifery model of care'—and what you see practiced in hospitals?
4. Thinking about your courses, are there specific topics or skills where you feel the curriculum is very strong? Conversely, are there areas where you wish you had more instruction?

---

3. Theme 2: Clinical Training and Skills Development (15-20 minutes)

Objective: To explore the reality of clinical placements and skills acquisition, adding context to quantitative gaps identified in the study.

Key Questions:

1. Let's turn to your clinical placements. Can you tell us about your experiences with getting hands-on practice?
2. Our preliminary discussions with stakeholders have pointed to challenges with supervision and students getting enough access to delivery cases during clinical training. We want to learn more about this from you. Can you describe the level of guidance and mentorship you receive from faculty and clinical staff during your placements?
3. How confident do you currently feel in your practical midwifery skills?
    - *Probe:* What skills do you feel most prepared to perform? Are there any skills where you feel you need more practice?
4. Interviews with graduates have suggested they sometimes feel unprepared for the counseling and patient education parts of their role. How well do you think your clinical training is preparing you for the communication aspects of midwifery care?

---

4. Theme 3: Career Concerns and Professional Future (15 minutes)

Objective: To explore students' career anxieties and expectations, which have emerged as a key theme in the ongoing qualitative data collection.

Key Questions:

1. Looking ahead to your graduation, what are you most excited about? What aspects of

your future career, if any, are you concerned about?
2. In our ongoing interviews, the topic of career paths and clear deployment strategies for midwives has come up frequently as a concern. Is this something you and your classmates discuss?
    - *Probe:* What are your thoughts or worries about finding a job and building a career as a midwife in Nepal?
3. The study has noted that there are very few formal government positions for midwives, and some graduates worry they will not be able to work in the role they were trained for. How does this information impact your motivation and your view of the profession?
4. What kind of support or incentives do you think would be most effective in encouraging students to complete their midwifery education and work in the profession, especially in areas where they are most needed?

---

5. Conclusion and Recommendations (10 minutes)

Objective: To summarize the discussion and gather student-led recommendations to inform the study's final conclusions.

Key Questions:

1. We have explored many important topics today. Based on your experiences, what is the single most important area that needs improvement in midwifery education in Nepal?
2. If you could give one piece of advice to the policymakers and educators who are working to improve midwifery in Nepal, what would it be?
3. Is there anything else you would like to share that would be important for our research team to know?

(Facilitator's Closing Script)

"Thank you all for your time and for sharing such open and valuable feedback. Your perspectives are a critical part of our ongoing research and will help ensure our final recommendations are grounded in the real experiences of students. We appreciate your contribution to this important work."
