# Supplementary material for: Alignment of Midwifery Education in Nepal With Global Standards and Essential Competencies From the International Confederation of Midwives: A Mixed‐Methods Study
Source: J Midwifery Womens Health. 2026 Apr 6;71(3):367–78. doi: 10.1111/jmwh.70096 (PMC13263916; doi:10.1111/jmwh.70096)
Supplement: Supplementary file 4 — Appendix S4. Observation Checklist [file JMWH-71-367-s004.docx]

**Appendix S4: On-Site Visit Checklist: Assessment of Midwifery Education Resources**

Site Visited: ____________________________________

Date: ____________________________________

Assessor: ____________________________________

*This checklist is derived from the ICM Global Standards for Midwifery Education, Category 5 (Resources), to assess the adequacy of the educational environment for pre-service midwifery training*.

**1. General Infrastructure**

This section assesses the fundamental physical space and support systems required for a functional learning environment.

| **Item** | **Assessment (Present & Functional)** | **Notes (Observations, Quality, Functionality)** |
| --- | --- | --- |
| **1.1** Adequate physical space for programme needs (classrooms, offices) | Y / N / Partial |  |
| **1.2** Functioning utilities (e.g., electricity, water, sanitation) | Y / N / Partial |  |
| **1.3** Overall maintenance and safety of infrastructure | Y / N / Partial |  |

**2. Learning Resources (Availability and Functionality)**

This section assesses the specific resources for theoretical and practical learning, including simulation, as specified in ICM Standard 5.1.

| **Item** | **Assessment (Present & Functional)** | **Notes (Observations, Quality, Functionality)** |
| --- | --- | --- |
| **2.1 Library Resources:** |  |  |
| 2.1.1 Access to current print texts and journals | Y / N / Partial |  |
| 2.1.2 Access to online literature and databases | Y / N / Partial |  |
| 2.1.3 Access to current clinical guidelines | Y / N / Partial |  |
| **2.2 Simulation Laboratory Resources:** |  |  |
| 2.2.1 Availability of anatomical models | Y / N / Partial |  |
| 2.2.2 Availability of simulation models (for skills practice) | Y / N / Partial |  |
| 2.2.3 Observation of simulation training (e.g., procedures, decision-making) | Y / N / Partial |  |
| **2.3 Classroom & Technical Resources:** |  |  |
| 2.3.1 Availability of current teaching aids (e.g., projectors) | Y / N / Partial |  |
| 2.3.2 Technical support for virtual/distance learning | Y / N / Partial |  |
| **2.4 Clinical Equipment Resources:** |  |  |
| 2.4.1 Availability of essential clinical equipment (e.g., Doppler monitors, birth supplies) | Y / N / Partial |  |
| 2.4.2 Functionality of essential clinical equipment | Y / N / Partial |  |

**3. Clinical Learning Environment**

This section assesses the quality and suitability of the clinical sites for student practice, aligning with ICM Standard 5.6.

| **Item** | **Assessment (Present & Functional)** | **Notes (Observations, Quality, Functionality)** |
| --- | --- | --- |
| **3.1** Quality of care at the site supports student competency | Y / N / Partial |  |
| **3.2** Observation of clinical practice (e.g., adherence to standards, respectful care) | Y / N / Partial |  |
| **3.3** Range of midwifery services available for student exposure |  |  |
| * Antenatal care | Y / N |  |
| * Labour and birth | Y / N |  |
| * Postnatal care | Y / N |  |
| * Newborn care | Y / N |  |
| * Family planning / post-abortion care | Y / N |  |

**4. Supervision and Practice Conditions**

This section assesses the support structure for students within the clinical environment, aligning with ICM Standards 3.8 and 5.6.

| **Item** | **Assessment (Present & Functional)** | **Notes (Observations, Quality, Functionality)** |
| --- | --- | --- |
| **4.1** Student-teacher ratios in clinical settings | (e.g., 1:8, 1:10) |  |
| **4.2** Quality of supervision provided to students (e.g., direct, indirect, feedback) | Y / N / Partial |  |
| **4.3** Primary supervisors are qualified midwives | Y / N / Partial |  |
| **4.4** Formal preparation/qualifications of clinical educators/preceptors | Y / N / Partial |  |
| **4.5** Practice conditions (e.g., workload, workflow) are conducive to student learning | Y / N / Partial |  |
